# Clinical and Radiographic Outcomes of the Wrap-Around Extension Block Pinning Technique for Bony Mallet Fingers

**DOI:** 10.7759/cureus.99816

**Published:** 2025-12-22

**Authors:** Raphael Israeli, Tomer Cohen, Gil Gannot, Dana Avraham, Amir Oron

**Affiliations:** 1 Orthopedic Surgery, Kaplan Medical Center, Rehovot, ISR; 2 Orthopedics, Clalit Health Services, Hamerkaz, ISR; 3 Medicine, The Hebrew University, Jerusalem, ISR

**Keywords:** bony mallet finger, distal interphalangeal joint, extension block pinning, finger fracture fixation, hand surgery, k-wire fixation, mallet fracture, percutaneous pinning, phalangeal fracture, wrap-around technique

## Abstract

Aims and objectives

Mallet finger fractures with substantial dorsal fragments often require surgical intervention, particularly when associated with distal interphalangeal joint (DIPJ) subluxation. We evaluated the clinical outcomes of a modified fixation method, the wrap-around extension block pinning technique, designed to enhance dorsal fragment compression and reduce K-wire migration.

Materials and methods

We conducted a retrospective cohort study of all consecutive patients treated with the wrap-around technique at a single tertiary hand surgery center over five years. Seventeen patients with a minimum follow-up of four months were included. Demographic, clinical, and radiographic data were analyzed. Outcomes were assessed using Crawford's criteria, and the results were benchmarked against historical controls (Çapkın et al., Jain et al., and the surgical and conservative subgroups of Yoon et al.). Graphical analyses were used to visually explore the potential trend between the mean cohort age and outcome distributions.

Results

Seventeen patients (mean age, 39 years) were analyzed. The combined excellent/good rate was 41.1% (7/17), and no postoperative complications occurred in this cohort (0%; 0/17), with no cases of hardware failure or loss of reduction. Comparative descriptive analysis suggested an inverse trend between cohort mean age and the proportion of excellent/good outcomes across the analyzed studies, with younger published cohorts demonstrating higher excellent/good rates. The outcome distribution in the current series most closely resembled that of the surgically treated subgroup in Yoon et al., which also included older patients and more complex fracture patterns.

Conclusion

The wrap-around extension block pinning technique provided exceptionally stable fixation without complications but did not yield superior functional outcomes compared with the historical results of standard extension block pinning. Our observations suggest that age, fracture severity, and immobilization duration may be more strongly associated with functional recovery than the specific fixation constructs. While further comparative studies are needed, the technique offers a reliable alternative when construct stability is a priority.

## Introduction

Mallet finger is a common injury of the terminal extensor mechanism, typically resulting from sudden flexion or, less commonly, hyperextension of the distal phalanx [1]. The lesion may present as a disruption of the terminal extensor tendon or as an avulsion fracture involving the dorsal base of the distal phalanx [2,3]. When a substantial bony fragment is avulsed, the distal interphalangeal (DIP) joint may demonstrate palmar subluxation, reflecting underlying articular instability and altering the preferred management approach for the injury [2].

While most soft-tissue mallet injuries are effectively managed with continuous extension splinting, bony mallet fractures involving more than 30% of the articular surface or those associated with DIP joint subluxation are generally considered indications for surgical treatment [2,4-6]. Surgical intervention is generally indicated in these patterns, as the loss of substantial articular support is associated with persistent volar subluxation and joint incongruity that are less likely to be adequately corrected with splinting alone [5]. Doyle’s classification further distinguishes these injuries, with type IV-B fractures characterized by involvement of more than 30% of the articular surface and type IV-C fractures presenting with volar subluxation of the distal phalanx, both of which are commonly treated operatively due to their inherent instability [7]. Operative fixation aims to restore joint congruity, minimize extensor lag, and prevent chronic deformities or degenerative disease progression [8]. Among the various surgical techniques described, extension block pinning has become one of the most widely adopted options because of its minimally invasive nature and reliable maintenance of reduction [6,9].

However, the classical extension block method is technically demanding, particularly when dealing with small or unstable dorsal fragments. The reported limitations include difficulty in maintaining dorsal fragment compression during wire insertion, loss of reduction, and K-wire migration risk [10-12]. To address these mechanical challenges, the wrap-around extension block pinning technique was developed as a modification of the Ishiguro technique [13]. Unlike the dorsal counterforce technique, which often requires additional leverage wires, the wrap-around modification utilizes the existing extension block wire to create a self-retaining compression loop. This construct is designed to improve stability by adding controlled dorsal compression and reducing the likelihood of wire displacement through a circumferential "wrap-around" configuration.

Despite its logical mechanical advantages, the clinical performance of this modification relative to that of established extension block methods has not been well defined. Previous literature on bony mallet fixation reports a wide range of outcomes, which appear to be influenced by patient age, fracture size, subluxation, and duration of immobilization [14-16]. Whether a modified construct can mitigate these biological and injury-related determinants remains unclear [17,18].

Therefore, this study aimed to evaluate the clinical and radiographic outcomes of the wrap-around extension block pinning technique in a retrospective cohort of patients treated at a single center and to compare these outcomes descriptively with contemporary published series. We sought to determine whether the hypothesized mechanical advantages translated into reliable clinical results. We emphasize that this comparison is descriptive only, as the study design precludes statistical testing for non-inferiority or superiority.

## Materials and methods

Study design and setting

This retrospective study reviewed patients treated for bony mallet finger using the wrap-around extension block pinning technique at Kaplan Medical Center, Rehovot, Israel. Eligible cases were identified from outpatient and inpatient medical records between January 2015 and January 2020. The study protocol was approved by the Institutional Helsinki Committee (No. KMC-0172-18). All procedures were performed by a single fellowship-trained hand surgeon to ensure consistency in the operative technique and postoperative management. Only patients with a minimum clinical follow-up of four months were included.

Patient selection and surgical indications

Patients were eligible for inclusion if they had a bony mallet finger with either (1) a fracture fragment involving >30% of the DIP joint articular surface or (2) volar subluxation of the distal phalanx. These injuries correspond to Doyle type IV-B and IV-C fractures [8]. Seventeen patients met the inclusion criteria and were included in the final analysis.

Surgical technique

All procedures were performed under digital nerve block anesthesia, without a tourniquet. The wrap-around extension block pinning technique is a modification of the standard extension block pinning technique [15]. First, a 0.8 mm K-wire was inserted at approximately 45° to the long axis of the finger, just proximal to the dorsal fracture fragment, and advanced into the head of the middle phalanx to serve as an extension block wire. A second K-wire (1.0-1.2 mm) was introduced longitudinally across the DIP joint to transfix the distal and middle phalanges.

**Figure 1:**
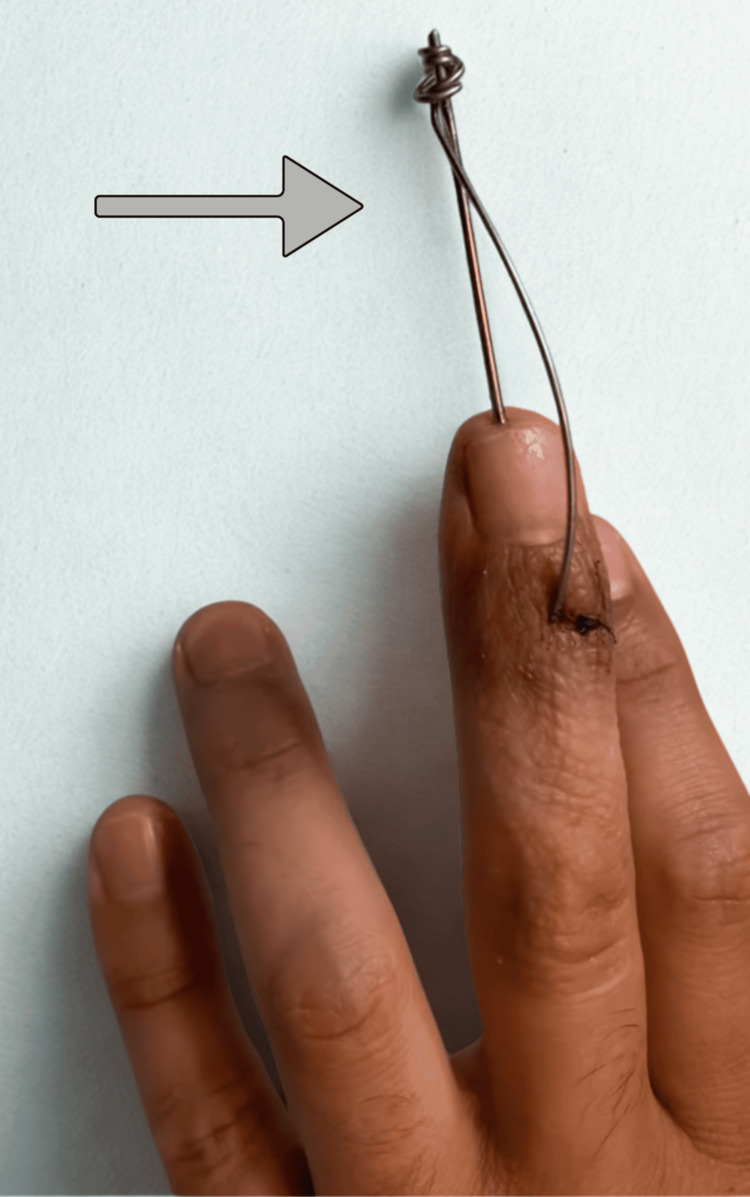
Postoperative clinical view of the wrap-around extension block pinning technique The construct shows the dorsal extension block wire bent and wrapped around the longitudinal transfixing wire. This configuration locks the extension block to prevent wire migration and maintains controlled compression of the dorsal fracture fragment.

After fluoroscopic confirmation of anatomic reduction, the dorsal fragment was compressed by bending the extension block wire around the longitudinal transfixing wire in a wrap-around configuration (Figure 1). This construct maintained dorsal compression of the fragment and stabilized the reduction of the fracture. When necessary, minor bending adjustments of the dorsal wire were made to decrease the soft tissue pressure. The final reduction and joint alignment were verified fluoroscopically, and the wire ends were cut and protected with padded dressings.

Postoperative management

Immediately after surgery, a standard dorsal extension splint was applied to protect the pin construct in all patients. Patients were permitted light functional use of the hand while avoiding strong gripping or other forceful activities. Active range-of-motion exercises of the proximal interphalangeal (PIP) joint were encouraged during the early postoperative period, and wrist and elbow motions were unrestricted. No standardized formal physical therapy protocol was utilized; instead, patients were instructed on a home exercise program. The dressings were kept clean and dry; if they became wet or contaminated, they were changed, and the pin sites were cleansed with alcohol. The hardware was routinely removed between four and five weeks postoperatively. Approximately one week after K-wire removal, the patients began both active and passive range-of-motion exercises of the DIP joint.

Outcome measures

Clinical and radiographic assessments were performed during routine follow-up visits. When an in-person evaluation was not feasible, patients were contacted by telephone and asked to provide video recordings demonstrating active motion of their affected DIP joint. The primary outcome measure was the functional result according to Crawford’s criteria (excellent, good, fair, and poor) [19]. Additional clinical data included the active DIP joint range of motion and degree of extension lag at the final follow-up. The radiographic outcomes included maintenance of reduction, joint congruity, and presence or absence of degenerative changes at the DIP joint. Complications were comprehensively defined to include any adverse event, ranging from superficial pin-tract irritation, minor skin breakdown, and unplanned clinic visits to major issues such as nail deformity, K-wire migration, recurrent subluxation or dislocation, and radiographic arthrosis.

Data analysis

Demographic and clinical variables were summarized descriptively using means, ranges, counts, and percentages. Outcome distributions according to Crawford's criteria were tabulated for the current cohort and compared descriptively with previously published series presented in the tables and figure. Regarding the graphical analysis of trends, no formal statistical correlation was performed due to the small sample size; thus, these visual comparisons are intended as exploratory only.

## Results

A total of 17 patients met the inclusion criteria and were included in the study. The cohort consisted of 10 men and seven women, with a mean age of 39 years (range, 16-64 years) (Table 1). The left hand was involved in 12 cases and the right hand in five cases. The little, middle, and ring fingers were affected in nine (52.9%), five (29.4%), and three (17.6%) patients, respectively. All patients completed a minimum follow-up period of four months. No patients were lost to follow-up, and no clinical or radiographic data were missing from the study.

**Table 1:** Patient demographics and clinical characteristics Data are presented as number (percentage) unless otherwise indicated. Age is expressed as mean (range).

Characteristic	Value
Total patients	17
Gender	
Male	10 (58.8%)
Female	7 (41.2%)
Age (years)	
Mean (range)	39 (16–64)
Hand involved	
Right	5 (29.4%)
Left	12 (70.6%)
Digit distribution	
Little finger	9 (52.9%)
Middle finger	5 (29.4%)
Ring finger	3 (17.6%)
Clinical timeline	
Follow-up duration	24 months
Hardware removal timing	4–5 weeks

Clinical outcomes

According to Crawford’s criteria, four patients (23.5%) achieved excellent outcomes, three (17.6%) had good outcomes, and eight (47.1%) had fair outcomes. No patient had poor outcomes. Therefore, the combined excellent/good rate was 41.1% (Table 2).

**Table 2:** Distribution of clinical outcomes according to Crawford's criteria The value n represents the number of patients. Data are presented as number (percentage). The combined excellent/good outcome rate was 41.1% (n = 7). Crawford’s classification criteria describe the degree of extension lag and flexion loss.

Crawford's grade	Criteria	Patients n (%)
Excellent	0° extension lag; full flexion	4 (23.5%)
Good	0-10° extension lag; full flexion	3 (17.6%)
Fair	11-25° extension lag; any flexion loss	8 (47.1%)
Poor	>25° extension lag	0 (0.0%)

Radiographic findings

Radiographic evaluation demonstrated maintained fracture reduction in all cases. No postoperative volar subluxation, dorsal fragment displacement, or recurrent malalignment was noted. No radiographic arthrosis of the DIP joint was observed on plain radiographs obtained at the final follow-up, within the limits of the available study period.

Complications

No postoperative complications occurred. Specifically, there were no cases of nail deformity, K-wire migration, recurrent dislocation, pin-tract problems, or malunion.

Comparative outcome data 

**Figure 2:**
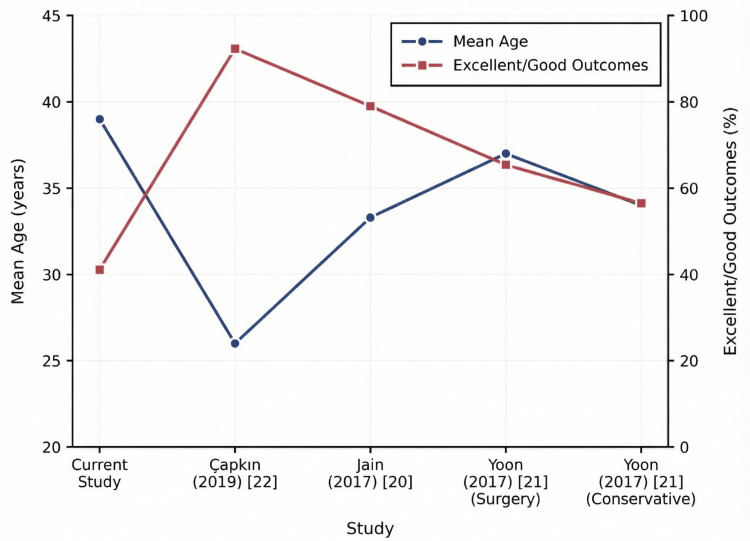
Relationship between mean cohort age and the proportion of excellent/good outcomes Dual-axis plot illustrating the mean patient age (left axis, blue line) and the percentage of patients achieving excellent or good Crawford’s outcomes (right axis, red line). Comparison includes the current cohort and data derived from Çapkın et al., Jain et al., and Yoon et al.

The outcome distributions from this cohort were compared descriptively with four published series using the data presented in Table 3. The excellent/good rate in the current study was 41.1%, which is lower than that reported by Çapkın et al. and Jain et al. and similar to that of the surgically treated subgroup reported by Yoon et al. [20-22]. Graphical analysis (Figure 2) illustrates the variation in outcome categories across the five cohorts and suggests an inverse trend between the mean cohort age and the proportion of excellent/good outcomes in the cohorts.

**Table 3:** Comparison of outcomes with previous studies The value n represents the number of patients. Data are presented as percentages calculated according to Crawford’s criteria, and mean age is presented in years. Comparative data are derived from Çapkın et al., Jain et al., and the surgical and conservative subgroups of Yoon et al.

Study	n	Mean age (years)	Excellent (%)	Good (%)	Fair (%)	Poor (%)
Current study	17	39.0	23.5	17.6	47.1	0.0
Çapkın et al. [22]	13	26.0	61.5	30.8	7.7	0.0
Jain et al. [20]	19	33.3	57.9	21.1	21.1	0.0
Yoon et al. (surgery) [21]	26	37.0	19.2	46.2	23.1	11.5
Yoon et al. (conservative) [21]	23	34.0	8.7	47.8	34.8	8.7

## Discussion

This study evaluated the clinical and radiographic performance of the wrap-around extension block pinning technique, a modification of the classic extension block pinning technique, designed to enhance dorsal fragment compression and minimize K-wire migration. Across the cohort, all patients maintained stable fracture reduction without postoperative volar subluxation, recurrent displacement, or radiographic arthrosis, reaffirming the capacity of percutaneous techniques to provide reliable structural stability for bony mallet fractures [8,13].

From a clinical standpoint, 41.1% of patients achieved excellent or good Crawford’s outcomes. While this rate is lower than some reported series, it must be weighed against the technique's safety profile. Compared with published series, the outcome distribution in the present cohort most closely resembled that of the surgically treated subgroup reported by Yoon et al. and differed from the more favorable distributions described by Çapkın et al. and Jain et al. [20-22]. These discrepancies highlight the variability in the reported results and may reflect differences in patient demographics and injury characteristics. In particular, the mean age of our cohort (39 years) was higher than that in some studies with superior outcomes. Younger individuals often demonstrate greater joint mobility and soft tissue adaptability, which may partly explain the outcome profile observed in this study [23,24].

The wrap-around modification was specifically designed to address the mechanical limitations inherent to standard extension block pinning, namely, the dorsal fragment control and K-wire stability [10,25]. Intraoperatively, the construct provided a predictable method for maintaining dorsal compression, with the wrap-around configuration effectively locking the extension block wire against the transfixing wire. The absence of wire migration or soft tissue complications in all 17 cases supports the mechanical reliability of this modification. These technical benefits are especially pertinent in fractures with fragile dorsal fragments, in patients with increased soft tissue tension, or in scenarios where precise adjustments to dorsal pressure are necessary to optimize reduction [12,25,26]. This zero-complication rate is an encouraging finding consistent with the construct's design, as standard extension block pinning is frequently associated with pin migration, skin irritation, or loss of reduction [6,14,15]. However, given the small sample size (n = 17), it is acknowledged that rare complications may not have been captured, and these results should be interpreted with caution.

Despite these mechanical strengths, the technique did not yield superior functional outcomes compared with the historical extension block series. This observation underscores an important concept: fixation method alone may not be the primary driver of functional recovery in bony mallet fractures [14,15,17,18]. Factors such as the initial injury pattern, articular fragment morphology, age-related joint stiffness, and postoperative immobilization duration likely play substantial roles [3,12,14,27,28]. Earlier cases in our series involved slightly longer immobilization periods, and prolonged immobilization is known to contribute to extension lag and reduced DIP arc of motion [1,3,29]. The interaction of biological, mechanical, and rehabilitation-related factors likely accounts for the wide variability in functional recovery reported in previous studies [8,12].

Clinical applicability

The primary advantage of the wrap-around extension block pinning technique is its safety profile and mechanical reliability. In this series, we observed a zero-complication rate, with no instances of pin migration, skin irritation, or reduction loss. While functional outcomes were not superior to historical controls [20-22], the enhanced stability provided by the circumferential loop makes this technique a valuable option for complex injuries, such as those with volar subluxation or tenuous dorsal fragments [2,26]. Furthermore, the rigid stability observed suggests that future protocols could incorporate earlier hardware removal (3-4 weeks) to mitigate stiffness without compromising fracture union.

Limitations

This study has inherent limitations. First, the retrospective design and small sample size (n = 17) precluded advanced statistical modeling; thus, the relationship between patient age and outcome remains descriptive. Second, the lack of a contemporaneous control group treated with the standard extension block method limits our ability to isolate the technique’s efficacy from other variables like rehabilitation protocols. Third, as this series represents our initial experience with the modification, a learning curve regarding immobilization duration likely influenced the functional results. Fourth, the follow-up period, although adequate for assessing union, was insufficient to fully evaluate long-term sequelae, such as post-traumatic osteoarthritis. Fifth, owing to the retrospective nature of the study, precise angular range-of-motion measurements (degrees of flexion and extensor lag) were not consistently recorded in the clinical notes for all patients; consequently, functional outcomes were assessed using the categorical Crawford’s classification rather than continuous angular variables. Finally, although no complications were observed, the limited sample size suggests that rare adverse events may not have been captured, and the zero-complication rate should be interpreted with caution.

## Conclusions

The wrap-around extension block pinning technique demonstrated reliable anatomic reduction and a zero-complication profile, with no instances of hardware failure, migration, or loss of reduction. While functional outcomes were comparable to, rather than superior to, historical extension block series, the technique’s mechanical robustness offers a distinct advantage for unstable fracture patterns where maintaining reduction is challenging. Consequently, this modification serves as a reliable option for maintaining reduction. Future prospective studies are warranted to determine if the construct's enhanced stability allows for accelerated rehabilitation protocols to potentially improve long-term range of motion.
